# Supplementary material for: Assessment of sexual dimorphism Desmodus rotundus (Chiroptera: Phyllostomidae), a rabies reservoir in Latin America
Source: bioRxiv. 2025 Feb 20:2025.02.17.638589. Preprint. [Version 1] doi: 10.1101/2025.02.17.638589 (PMC11870455; doi:10.1101/2025.02.17.638589)
Supplement: Supplement 1 [file NIHPP2025.02.17.638589V1-supplement-1.pdf]

bioRxiv preprint doi: https://doi.org/10.1101/2025.02.17.638589; this version posted February 20, 2025. The copyright holder for this preprint (which was not certified by peer review) is the author/funder, who has granted bioRxiv a license to display the preprint in perpetuity. It is made available under aCC-BY 4.0 International license.

S1. Alternative Language Abstract (Spanish)

S2. Dataset: Morphometric Measurements of *Desmodus rotundus* Specimens
